# An Intelligent Control Model of Credit Line Computing in Intelligence Health-Care Systems

**DOI:** 10.3389/fpubh.2021.718594

**Published:** 2021-09-10

**Authors:** Rong Jiang, Wenxuan Wu, Yimin Yu, Feng Ma

**Affiliations:** ^1^Institute of Intelligence Applications, Yunnan University of Finance and Economics, Kunming, China; ^2^Key Laboratory of Service Computing and Safety Management of Yunnan Provincial Universities, Kunming, China; ^3^Kunming Key Laboratory of Information Economy & Information Management, Kunming, China; ^4^School of Information, Yunnan University of Finance and Economics, Kunming, China

**Keywords:** machine learning, biomedical computing, intelligence healthcare, privacy security, intelligent access control, credit line

## Abstract

Technologies such as machine learning and artificial intelligence have brought about a tremendous change to biomedical computing and intelligence health care. As a principal component of the intelligence healthcare system, the hospital information system (HIS) has provided great convenience to hospitals and patients, but incidents of leaking private information of patients through HIS occasionally occur at times. Therefore, it is necessary to properly control excessive access behavior. To reduce the risk of patient privacy leakage when medical data are accessed, this article proposes a dynamic permission intelligent access control model that introduces credit line calculation. According to the target given by the doctor in HIS and the actual access record, the International Classification of Diseases (ICD)-10 code is used to describe the degree of correlation, and the rationality of the access is formally described by a mathematical formula. The concept of intelligence healthcare credit lines is redefined with relevance and time Windows. The access control policy matches the corresponding credit limit and credit interval according to the authorization rules to achieve the purpose of intelligent control. Finally, with the actual data provided by a Grade-III Level-A hospital in Kunming, the program code is written through machine learning and biomedical computing-related technologies to complete the experimental test. The experiment proves that the intelligent access control model based on credit computing proposed in this study can play a role in protecting the privacy of patients to a certain extent.

## Introduction

Medical big data (1) is a branch of big data in the field of biomedicine. It refers to the data related to life, health, and medical care generated in activities related to human health, mainly from intelligent medical systems such as clinical data, hospital, operation, biomedical research, disease prevention and control, health protection and food safety, public health and health management data, health care and other aspects (2). In these massive amounts of data, there are opportunities. If the data generated by smart medical care can be flexibly called after biomedical calculations, data pressure can be converted into data advantage (3, 4).

In terms of biomedicine, individual users have become an important source of data. The private information generated by smart medical treatment often means unspeakable pain for individuals. The leakage of such negative information has become a huge hidden danger in the era of big data (5). In the past, most patients maintained their personality and dignity through self-forgetting and the privacy of medical institutions (6). Nowadays, the ubiquitous smart medical equipment and cloud storage and cloud computing functions, such as placing users in a transparent glass room. Our every move may be recorded, and the electronic health records generated by the widely used smart medical system and smart medical equipment make it difficult for patients to hide their privacy. According to a security report released by Trust wave, more than 90% of the investigators believe that there are more and more cyberattacks against the medical field, but the budget for protecting sensitive patient information is <10% (7). Once the criminals steal medical data, they can easily learn the name, home address, contact information, test report, diagnosis results, and even medical insurance and other important information of the patient, and use this to falsify the data to defraud or purchase medical equipment. Therefore, the consequences of data theft in the medical field are very serious. More than two million people in the United States will become victims each year. The loss caused by this is as high as $13,500, and it will take hundreds of hours to solve this problem. In 2015, the social security system became the hardest hit area for personal information leakage, etc. (8). These incidents seriously violated the privacy and legal rights of users. At present, both the public and the government have begun to pay attention to personal privacy issues in medicine (9). In the United States, electronic health data are also being prepared for an online transformation. Dosia (a non-profit coalition of major employers), Google Health, Microsoft Health Vault, and other network services are driving this transformation. These services are seeking expanded role in the United States health-care system that values 21,000 dollars (10).

With China's accession to the WTO and the acceleration of social information, whether it has a fully functional intelligent medical system, it has become an important indicator to measure the comprehensive strength of a hospital (11, 12). A perfect hospital information system (HIS) includes outpatient management, hospitalization management, drug management, multiple subsystems, such as electronic medical records and financial management. The high integration between the various subsystems improves the overall operating efficiency of the hospital, improves the medical environment of the patient, and at the same time provides data-driven support for the management of hospital, clinical, etc. The electronic medical record system (13) includes the electronic medical record of the doctor and nurse. The main function is to save the medical records of the patients electronically. It not only includes the medication information of the patient, but also includes the treatment record, laboratory and examination records, and other information of the patient. The doctor is giving the past medical records and medical history of the patient during treatment. It can more accurately analyze the condition of the patient and treat the patient (7). However, due to the use of HIS, doctors can access a large amount of medical information, and the resulting medical problem of privacy leakage is also very tricky. When the system security and data security are not guaranteed, the intelligent medical system is fragile, which will not only cause great troubles for medical work, but also greatly reduce the prestige of the intelligent medical system (14).

Given the medical privacy leakage risk arising from the widespread use of intelligent medical systems today, this study proposes an access control model based on credit line calculations for intelligent medical systems. In this model, when doctors use the intelligent medical system to diagnose patients, they use historical records to calculate credit lines, and dynamically restrict doctors' access rights based on their credit capabilities. Don't give unnecessary permissions, and will not affect the normal work of doctors, try to comply with the A principle (15). The main steps of model realization are as follows:

1. Through similarity function calculation, the results obtained by the mathematical method can be used to describe whether the inquiring behavior of the doctor is reasonable.
2. The appropriate weight calculation method is used to obtain the weight value so that the unreasonable behavior of the doctor is easy to lead to the decline of the credit limit, but the reasonable behavior of the doctor will not affect his trust limit.
3. According to the credit limit of the user, match the corresponding trust interval to achieve the ability to limit the access authority of the user. Doctors with a high credit limit will become larger and larger. On the contrary, doctors with a low credit limit will become smaller and smaller, until it is lower than the credit line threshold, and the visit is forbidden. In the existing model, doctors select medical records based on randomly assigned work goals, or medical records are selected based on the work goals selected by the doctors themselves, and this study defines that the doctors may not necessarily choose an honest work goal based on the preliminary examination information of the patient. In addition, the doctor will give a more accurate final diagnosis only after checking the medical records. The model can properly describe the real diagnosis process of the doctor, and it is more in line with the actual situation.

The contributions of this study are as follows:

1. Some contents have been added to the doctor behavior model in the study (16), which improves the performance of the model in screening curious doctors.
2. In a relatively mature intelligent medical system, the concept of the credit line is introduced as the carrier of medical trust computing.
3. After comparison, more appropriate trust calculation and weight calculation methods are selected to achieve the effect of using historical records to restrain the behavior of doctors and reduce the risk of privacy disclosure in the medical field.

## Related Works

If divided by authorization strategy, the access control model can be divided into the following: traditional access control model (DAC/MAC), role-based access control (RBAC) model (17), task and workflow-based access control (TBAC) model (18), task-based and role-based access control (TRBAC) model, etc. (19). RBAC model permissions are associated with roles, and users become members of corresponding roles, which greatly simplifies the management of permissions. However, the RBAC model cannot be directly used for more complex forms of access control (20). Goyal et al. (21) proposed an attribute-based mechanism to protect data and avoid setting data owner rules. However, the main disadvantage of using attribute-based methods is that it will bring a high workload to the user side. For most ordinary users, with limited knowledge of rules or strategy design, creating a complex data access mechanism in a medical environment is an arduous task (22). The workload brought by traditional access control obviously cannot adapt to the situation of massive data in the context of big data.

Health and medical big data are important basic strategic resources of the country. Traditional database achieves security and privacy protection through data granularity-based security control, but the operation of big data still lacks effective security protection measures (23). The realization technology of medical big data information security includes access control and password technology. Data privacy implementation technologies include obfuscation, anonymity, differential privacy, and encryption (16). At present, the prominent problems in the use of HIS medical data mainly include the following:

- Security issues: dynamic permissions are granted. The existing medical information system does not consider the wishes of patients, and the scope of medical data that doctors can access is not detailed enough. According to the actual needs of doctors, there is little research on medical information systems that dynamically grant data access rights to achieve fine-grained data access.
- Data sharing issues: Doctors and researchers have strict restrictions when accessing and sharing medical data (24).

The important issue studied in this study is access control, with the focus on protecting information from unauthorized access (25). Wang et al. (26) designed a secure authentication algorithm to limit the access rights of access objects in the electronic medical record (EMR) system. Zhu et al. proposed a user-friendly, easy-to-manage, attribute-based access control (ABAC) for cloud storage services in 2015. This mechanism defines the priority of attributes and refines the granularity of data access control in the cloud environment (27). Liu et al. (28) is based on the trust-based access control model, which combines dynamic hierarchical fuzzy systems with trust evaluation, layered the attributes related to trust in the cloud manufacturing environment, and proposed a multi-attribute fuzzy trust evaluation access control scheme. Gao (29) built a flexible dynamic access control model to make up for the lack of static policies, making the original role, the static authorization access of permissions is transformed into a model that can dynamically authorize users. Zhang and Zhou (30) to solve the problems of access resources insufficient flexibility and preset allocation of permissions in the traditional role-based access control system, improved compatibility of access control, refine the granularity of access control, and propose a dynamic multilevel access control model based on trust. The static role and dynamic trust degree of the user obtain the corresponding authority authorization (31). Based on the traditional free access control (DAC) and RBAC model, a context-sensitive access control method is proposed, which strictly follows regulatory and technical standards in the health-care field to ensure authorized access (32). The literature found that existing symmetric and asymmetric encryption technologies have complex key management and certificate management problems. In response to these problems, the policy-based access control (PBAC) model and the purpose of joining conditions are proposed. IBE encryption technology medical data can access control scheme. Yang et al. (33) proposed a privacy protection medical big data system with adaptive access control. Through a new dual access control mechanism, the mechanism has adaptive capabilities for both normal and emergency medical data access. In summary, although various access control models have been expanded by previous researchers, yet studies on trust computing and access control models in the field of intelligent medical research are inadequate (34). The authorization method for access control in the process of diagnosis by doctors and treatment is relatively simple and restrictive. The problem of insufficient binding still exists.

Therefore, it is more necessary to explore a dynamic trust computing method and access control scheme, which are more suitable for specific occasions of medical access, and dynamically adjust the permissions of doctors to access medical resources through the results of trust computing, to improve the privacy protection performance of the model.

## Model Design

Based on the research of a large number of existing intelligent medical systems (35), this section presents the following behavior model which is more suitable for the actual situation. Taking the process of diagnosis by doctors and treatment of patients as the research object, the diagnosis and treatment behavior of doctors is abstracted into a model from the three aspects of examination information of doctors, access of doctors to medical data, and the diagnosis results are given. Then, the definition of credit limit, correlation calculation, and weight determination method are given.

### Doctor Behavior Model

**Figure 1:**
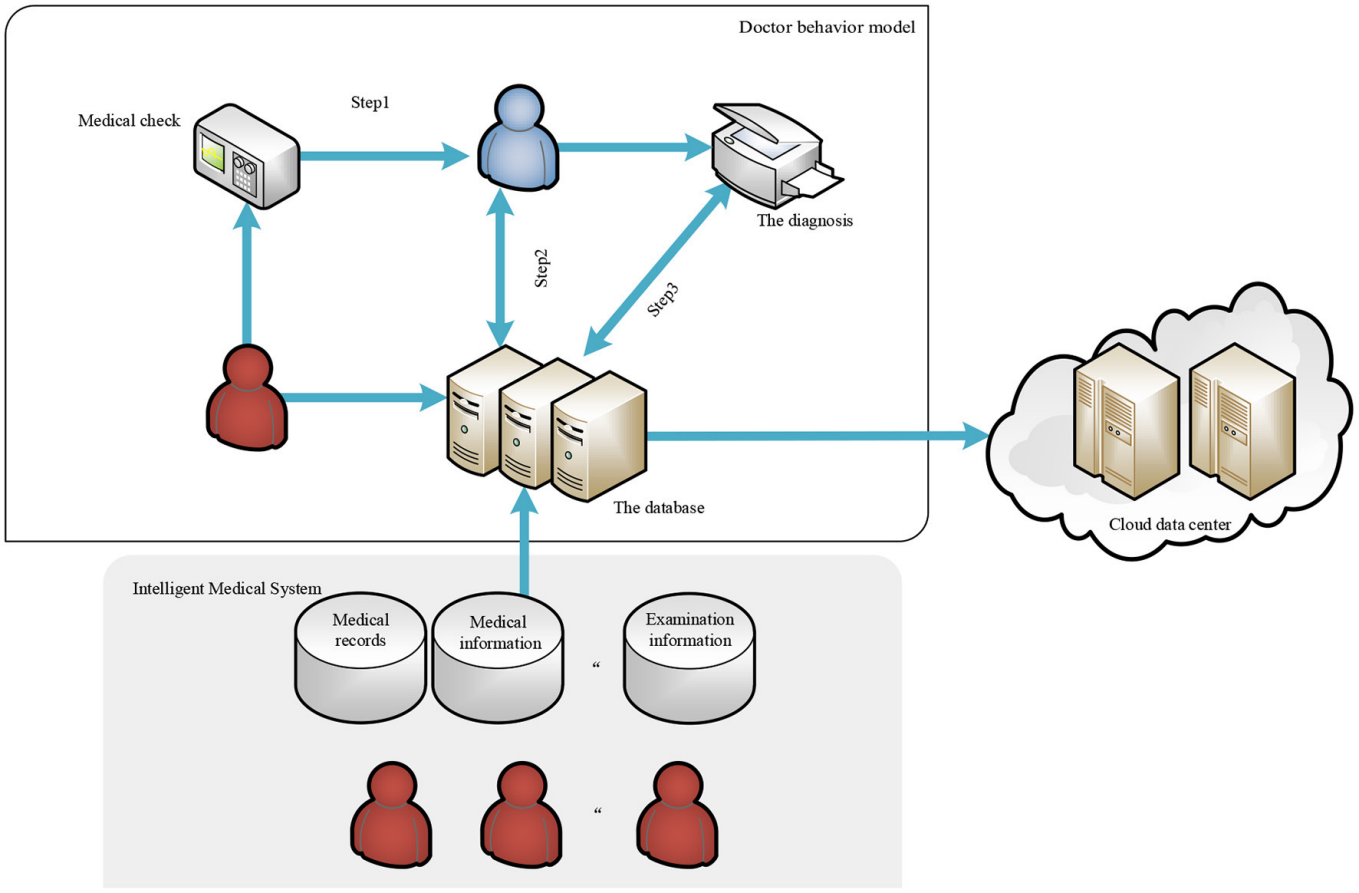
Doctor behavior model.

This is shown in Figure 1, for the diagnosis and treatment process of each patient, we call it a task. In the task, the diagnosis by doctor and treatment steps are generally the following: first browse the basic information of the patient, such as name, age, and past medical history. If the patient has a medical history in the hospital, the doctor can check the past examination items and results of the patient through the HIS database. Then, the patient will receive new test results according to the arrangement of the doctor, whose arrangement is also stored in the HIS database. The doctor can view the test results of the patient and certain related medical records (such as medical imaging data of other similarly diagnosed patients in the database, etc.) to obtain the final diagnosis for the patient. The medical data in the HIS database that doctors can view involve some sensitive information about the patient, but considering moral factors and the cost of leaks, hypothetical model doctors in China will not disclose any information about their patients. Based on the above behavior model, the behavior of privacy leakage of curious doctors will occur in the following three steps:

Step 1. The correlation between the examination information of the patient and the initial diagnosis given by the doctor is low. For example, the results of the examination of a patient can directly indicate that the patient is unlikely to have an infectious disease. However, the doctor still gave a preliminary diagnosis that the patient may have an infectious disease, and then consulted the relevant medical records of the patient with infectious disease based on the false preliminary diagnosis.
Step 2. Suppose that the doctor gave a correct preliminary diagnosis consistent with the examination information in Step 1, but inquired about unnecessary medical records when accessing the medical records based on the preliminary diagnosis.
Step 3. Assume that the doctor is operating normally in Step 1 and Step 2, but the final diagnosis has a low correlation with the medical records queried. It is suspected that the doctor had accessed unnecessary medical records.

Give the formal description of the symbol as follows, and abstract the process:

*E*: A collection of examination information;
*P*: A collection of primary diagnoses;
*F*: A collection of final diagnoses;
*R*: A collection of medical records.

*S*_1_:*E, P* → [0, 1]: Define the correlation function between inspection information and preliminary diagnosis, *e* ∈ *E, p* ∈ *P*, where the return value of the function reflects the degree of correlation between the two in a certain diagnosis and treatment process.
*S*_2_:*P, R* → [0, 1]: Define the correlation function between the initial diagnosis and medical records, *p* ∈ *P, r* ∈ *R*, where the return value of the function reflects the degree of correlation between the two in a certain diagnosis and treatment process.
*S*_3_:*R, F* → [0, 1]: Define the correlation function between medical records and the final diagnosis, *r* ∈ *R, f* ∈ *F*, where the return value of the function reflects the degree of correlation between the two in a certain diagnosis and treatment process.

### Trust Attribute System

The existing trust system for access control is relatively single, usually divided into direct trust and indirect trust. In the context of diagnosis and treatment by doctors, this trust model cannot accurately assess the credibility of behavior of doctors. Doctors have extensive access to patients and medical records in intelligent health-care systems, but there is a lack of effective direct trust between each doctor or between patients and other doctors (who do not diagnose themselves). According to the behavioral characteristics of diagnosis and treatment by doctors and the particularity of the structure of medical resource system, indirect trust is not considered in the trust attribute, but only the historical visit records of doctors will directly affect their credit line.

The concept of credit originates from the financial field and refers to the funds provided by banks to non-financial users, including but not limited to various businesses, such as loans. The credit line means the highest credit value given to users by the bank after calculation and evaluation during the credit period.

**Figure 2:**
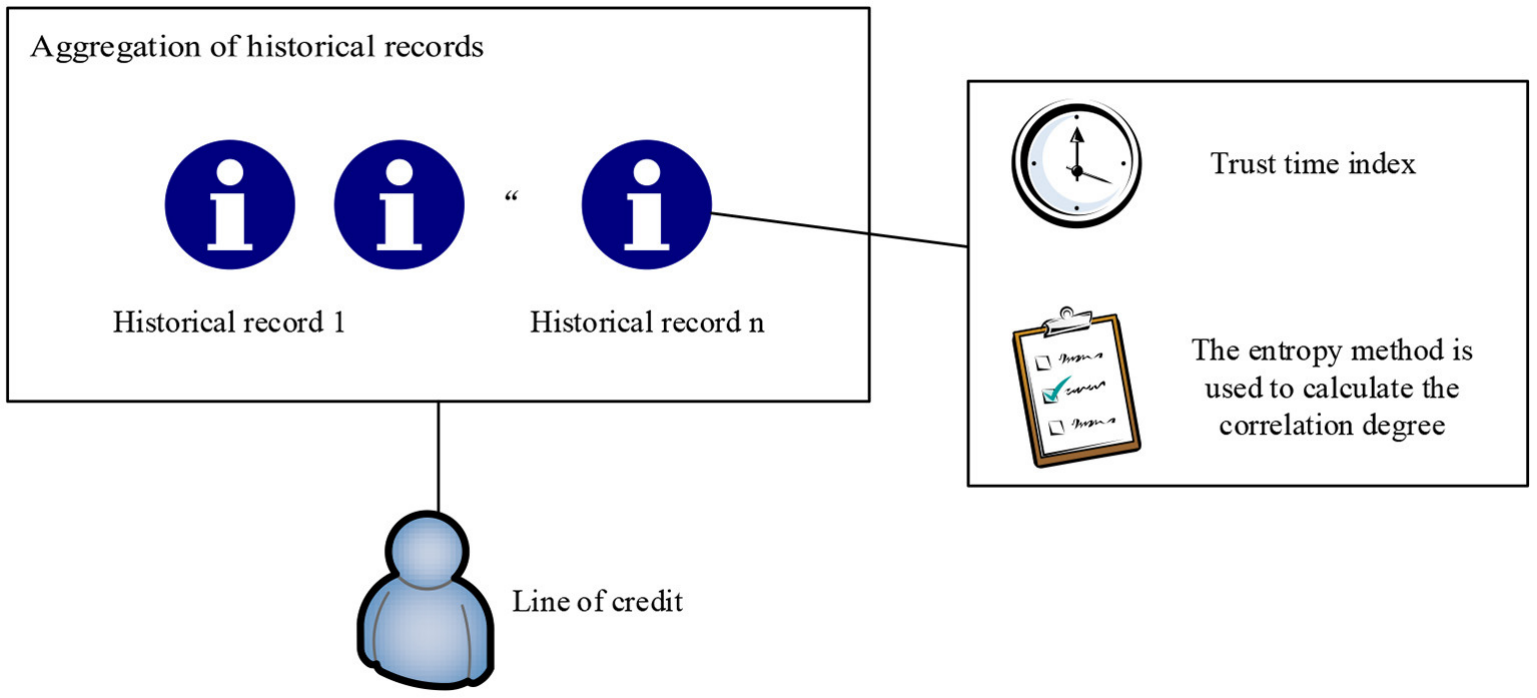
Trust calculation process.

This study introduces the concept of credit line in the intelligent medical system, and redefines it as a comprehensive evaluation of the history records, access behavior, and other factors of medical information system, and calculates and grants credit line of the doctor user for overdraft use. The credit limit is calculated by reading the history of the doctor through HIS. The continuous integrity behavior record of the doctor can help increase the credit limit, and high-risk behaviors will lead to a reduction in the credit limit, thereby realizing dynamic access control to medical data. The history record includes two sub attributes of the trust time window and operational relevance. This is shown in Figure 2.

### Correlation Calculation

In the intelligent medical system, doctors use electronic medical records to record the medical treatment of patients during the medical treatment process. This study introduces the International Classification of Diseases (ICD) as the code used by doctors in the diagnosis of electronic medical records. Suppose a certain disease in the electronic medical record is represented by an ICD code. Afterward, you can use the element group to write *a*_1_, *a*_2_, *a*_3_, …, *a*_*n*_, the elements representing the disease at each location are divided into different subcategories according to the ICD code and expressed as *a*_*i*1_, *a*_*i*2_, *a*_*i*3_, …, *a*_*in*_, where n represents the number of subcategories of the disease. To calculate the similarity, prepare to construct the initial judgment matrix EQ from the diseases in the medical records as follows:

(1) $$\begin{array}{l} EQ=\left\{\begin{array}{ccc} a_{11} & \ldots & a_{1n}\\ \ldots & \ldots & \ldots \\ a_{n1} & \ldots & a_{nn} \end{array}\right\} \end{array}$$

The three steps involved in diagnosis process by a doctor have different risks of privacy leakage. This section focuses on the calculation method of the correlation function. There are many methods to measure similarity (36), and the distance measurement is Minkowski distance, Euclidean distance, Manhattan distance, Hamming distance, etc. Commonly used similarity coefficients include cosine similarity, Pearson correlation coefficient, Jaccard correlation coefficient, etc. The traditional methods for measuring the similarity of two individuals are commonly used. Cosine similarity is defined as follows: regarding user information as an n-dimensional vector, the similarity is calculated as the cosine of the angle between the vectors. The similarity between individual i and j is recorded as shown:

(2) $$\begin{array}{l} \text{sim}\left(i,j\right)=\cos\left(\vec{i},\vec{j}\right)=\frac{\vec{i}\cdot \vec{j}}{\left||\vec{i}||*||\vec{j}|\right|} \end{array}$$

$\vec{i}\cdot \vec{j}$ is the inner product and $\left||\vec{i}||*||\vec{j}|\right|$ is the vector product.

In addition to cosine similarity, there is also correlation similarity. The similarity is measured by calculating the correlation coefficient between i and j of the item. Determine the common user set U of i and j, and the correlation similarity is defined as follows:

(3) $$\begin{array}{l} \text{sim}\left(i,j\right)=\frac{\underset{u\in U}{\sum }\left(R_{ui}-R_{i}\right)\left(R_{uj}-R_{j}\right)}{\sqrt{\underset{u\in U}{\sum }{\left(R_{ui}-R_{i}\right)}^{2}}\sqrt{\underset{u\in U}{\sum }{\left(R_{uj}-R_{j}\right)}^{2}}} \end{array}$$

The similarity is obtained by using cosine or correlation similarity, because the medical data category base is relatively large, and the data are dense, and the wrong conclusion with high similarity is obtained. When calculating the similarity, the Jaccard similarity coefficient is introduced to calculate the privacy leakage risk of the doctor in each step of the diagnosis process. The Jaccard similarity coefficient is also called the Jaccard index, which is used to compare the similarity and difference statistics of a limited sample set (37). Assume that sets I and F are the initial diagnosis and the final diagnosis is described using ICD codes. Each code contains n public attributes, which indicate the category and subcategory of the disease. Each attribute in the code consists of a number or letter. To facilitate the calculation, the number will be represented by the set of 0 and 1. The Jaccard index can be written as J (I, F). The definition of the Jaccard index is as follows (38):

(4) $$\begin{array}{l} J\left(I,F\right)=\frac{I⋂F}{I⋃F} \end{array}$$

Define that when I=F=Ø, J(I, F) = 1, the value range is [0,1], the larger the J value, the greater the similarity between the two samples. From this, the Jaccard distance can be obtained, and dJ (I, F) is used to represent the difference between the two samples:

(5) $$\begin{array}{l} {\text{d}}_{J}\left(I,F\right)=1-J\left(I,F\right)=\frac{\left|I⋃F|-|I⋂F\right|}{\left|I⋃F\right|} \end{array}$$

Taking the stomatology department as an example, suppose that the initial diagnosis given by a doctor is periodontitis. Define M11 as the number of ones in both I and F; M01 is the number of attributes of F that are 1 when the attribute of the set I is 0; M10 is the number of attributes of F in the set I that is 0 when the attribute is 1; M11 is the number of attributes of the set I and F that are 1. According to the above assumptions, the calculation method of Jaccard index and Jaccard coefficient can be obtained as follows:

(6) $$\begin{array}{l} M_{11}+M_{01}+M_{10}+M_{00}=\text{n} \end{array}$$

(7) $$\begin{array}{l} J=\frac{M_{11}}{M_{11}+M_{01}+M_{10}} \end{array}$$

(8) $$\begin{array}{l} {\text{d}}_{J}=\frac{M_{01}+M_{10}}{M_{11}+M_{01}+M_{10}} \end{array}$$

According to the analysis of the above doctor behavior part, the doctor can directly contact the medical data of the non-attending patient during the diagnosis process, which is a high-risk reason for privacy leakage. To avoid a single error in the similarity calculation, cross-entropy is then used (39) to introduce the calculation of the similarity between two random variables.

Entropy is the expected value of the amount of information (40). Assuming that there is a random variable x with a value range of set X, its probability distribution function can be expressed as p(*x*) = Pr(*X* = *x*), *x* ∈ *X*, and defines the amount of information as *I*(*x*_1_) = −lg(*p*(*x*_1_)), the greater the probability of an event, the more *p*(*x*_1_) larger, the smaller the amount of information it carries (41). In the extreme case *p*(*x*_1_) = 1, the amount of information is equal to zero, which means that when the probability of an event happening is 100%, then the occurrence of this event will not introduce too much information. When we know the amount of information to measure the uncertainty of the occurrence of an event, we can calculate the expectation (*E*[*I*(*x*)]) for the additional information brought by all possible results, and the entropy can be defined as follows:

(9) $$\begin{array}{l} H\left(X\right)=Ep\left[\text{ lg }p\left(x\right)\right]=-\sum x\in Xp\left(x\right)\text{ lg }p\left(x\right) \end{array}$$

According to the diseases and symptoms covered by ICD coding statistics, the average information required for each preliminary diagnosis (disease) was calculated as the threshold value. Suppose that the preliminary diagnosis obeys a random distribution p, and the interview records of a doctor obey a random distribution q. Then, cross-entropy is introduced to calculate the similarity degree of p and q. The expectation obtained according to the distribution p is *H (P)*. For the diagnosis process of doctors, the access records are discrete variables, and the p distribution is represented by the q distribution, which is called the cross-entropy.

(10) $$\begin{array}{l} H\left(\text{p}\right)=\underset{i}{\sum }p\left(i\right)*\text{ lg }\frac{1}{p\left(i\right)} \end{array}$$

(11) $$\begin{array}{l} H\left(p,q\right)=\underset{i}{\sum }p\left(i\right)*\text{ lg }\frac{1}{q\left(i\right)} \end{array}$$

Assuming that p of a disease can be expressed as [1, 0, 0][1, 0, 0], and q obtained by a doctor A's visit to the historical record is [0.5, 0.4, 0.1][0.5, 0.4, 0.1], then according to the calculation method of cross-entropy of formula (11), the cross-entropy between the visit behavior of doctor in the process of diagnosis and the initial diagnosis given by doctor A can be obtained as follows:


$$\begin{array}{l} \begin{array}{l} H\left(p=\left[1,0,0\right],q=\left[0.5,0.4,0.1\right]\right)\\ =-\left(1*\text{ lg }0.5+0*\text{ lg }0.4+0*\text{ lg }0.1\right)\\ \approx 0.3H\left(p=\left[1,0,0\right],q=\left[0.5,0.4,0.1\right]\right)\\ =-\left(1*\text{ lg }0.5+0*\text{ lg }0.4+0*\text{ lg }0.1\right)\\ \approx 0.3 \end{array} \end{array}$$

If the interview record Q of Doctor B with the same preliminary diagnosis is[0.8, 0.1, 0.1][0.8, 0.1, 0.1], then, the cross-entropy between the visit and the preliminary judgment of Doctor B in the process of this diagnosis is follows:

$$\begin{array}{l} \begin{array}{c} H\left(p=\left[1,0,0\right],q=\left[0.8,0.1,0.1\right]\right)\\ =-\left(1*\text{ lg }0.8+0*\text{ lg }0.1+0*\text{ lg }0.1\right)\\ \approx 0.1H\left(p=\left[1,0,0\right],q=\left[0.8,0.1,0.1\right]\right)\\ =-\left(1*\text{ lg }0.8+0*\text{ lg }0.1+0*\text{ lg }0.1\right)\\ \approx 0.1 \end{array} \end{array}$$

It can be seen from the calculation results that cross entropy value of Doctor B is small, that is, the operational correlation is higher. If the threshold value of this disease is known to be 0.2, then, it can be concluded that Doctor B is accessing medical data safely, and Doctor A is suspected of a large privacy breach.

To calculate the accuracy of similarity, two calculation methods, namely, Jaccard coefficient and cross-entropy, were used to calculate the correlation degree of the diagnosis and treatment process. The final formula for calculating the correlation degree of the diagnosis and treatment process was as follows:

(12) $$\begin{array}{l} S=\frac{\left(1\text{+}{\text{d}}_{1}\right)\alpha }{2H_{1}d_{1}}\text{+}\frac{\left(1+d_{2}\right)\beta }{2H_{2}d_{2}}\text{+}\frac{\left(1+d_{3}\right)\gamma }{2H_{3}d_{3}}\left(\alpha \text{+}\beta \text{+}\gamma =1\right) \end{array}$$

Because the weight cannot simply be given a definite value, it is determined by the vague advice given by experienced experts.

A review of the relevant literature and consultation with medical professionals has been discussed as follows:

Hypothesis A: To obtain certain medical records, a curious doctor falsifies the information of the primary diagnosis that does not match the inspection information, thereby, rationalizing the second step. However, even if qualified doctors encounter patients with special circumstances, they will not make a preliminary diagnosis with a correlation below the threshold based on the examination information of the patient. Therefore, the rules at this stage are very strong. Once the initial diagnosis is wrong, the correlation between Step 2 and Step 3 is normal, and the risk of leakage of medical record privacy is relatively high. Therefore, the weight corresponding to Step 1 needs to be relatively large. In this case, even if a curious doctor performs normal operations in Step 2 and Step 3, the credibility of the calculation will be greatly reduced.
Hypothesis B: If a qualified doctor diagnoses a patient with rare symptoms, the doctor needs to refer to more medical records to determine what disease the patient has. At this time, Step 1 is normal and the correlation of Step 2 is decreased, but according to medical records, the final judgment Step 3 should also be normal. Therefore, during the diagnosis, the weight of Step 2 can be appropriately relaxed, so that doctors can have a larger space for resource selection, and the diagnosis process of doctors in complicated cases will not be restricted.
Hypothesis C: If a curious doctor tries to imitate the behavior of an ordinary doctor in Hypothesis B, the curious doctor will naturally give a final diagnosis with low relevance to the medical record. Assuming that in the context of the medical environment, all doctors will perform their duties. A patient will not be diagnosed by only one doctor, so curious doctors will not insist on making a wrong final diagnosis to steal medical data. Therefore, in this case, the conclusion of the doctor based on a large number of irrelevant medical records will be less relevant to the initial diagnosis given by malicious intent.

Therefore, to distinguish between hypothesis B and hypothesis C, the weight of S3, namely γ, should also be large.

According to the above hypothesis analysis, in each step, appropriate weights can provide doctors with a certain space for fault-tolerant visits or special situations requiring additional resources and can also effectively screen behaviors of curious doctors. Therefore, it is necessary to introduce appropriate weight determination technology. Xu and Zhou (42) further developed the maximum score deviation (MSD) method to obtain the weight of each index. The principle of the MSD method is that when multiple experts evaluate the evaluation factors, the higher the similarity with the evaluation of other experts, the less weight should be given. In theory, if two experts give exactly the same assessment because it does not help to draw consensus from the disagreement, the weight can be set to zero. For each expert *PF* · *p*_*i*_, we introduce a function *D*_*ki*_(*x*) to represent the scoring deviation between the evaluated step and the remaining steps:

(13) $$\begin{array}{l} D_{\text{ki}}\left(x\right)=\overset{N}{\underset{t=1}{\sum }}|s\left(h_{ki}\right)w_{k}-s\left(h_{kt}\right)w_{k}| \end{array}$$

where h_*ki*_ and h_*kt*_ are hesitation probability fuzzy numbers, *S*(*x*) is a scoring function, and *w*_*k*_ is the weight of expert *PF* · *p*_*i*_ *i, t* = 1, 2, …, *N* and *k* = 1, 2, …, *K*.

Thus, the total score deviation for all the steps evaluated by expert *PF* · *p*_*i*_ can be expressed as *D*_*ki*_(*x*) follows:

(14) $$\begin{array}{l} D_{k}\left(x\right)=\overset{N}{\underset{i=1}{\sum }}D_{ki}\left(x\right)=\overset{N}{\underset{i=1}{\sum }}\overset{N}{\underset{t=1}{\sum }}|s\left(h_{ki}\right)w_{k}-s\left(h_{kt}\right)w_{k}| \end{array}$$

To obtain the optimal weight vector, since the general weight vector meets the normalization in cognition of people, Zhou Wei introduced constraint condition Equation (15) based on Wang (43), transformed *w*_*k*_ into $\bar{w_{k}}$ through Equation (16), and obtained the weight vector $\bar{\text{w}}=\left(\bar{w_{1}},\bar{w_{2}},\ldots ,\bar{w_{k}}\right)$. In this study, a developed MSD method was adopted.

(15) $$\begin{array}{l} \overset{k}{\underset{k=1}{\sum }}{\left(w_{k}\right)}^{2}=1 \end{array}$$

(16) $$\begin{array}{l} \bar{{\text{w}}_{k}}=\frac{w_{k}}{\sum_{k=1}^{k}w_{k}} \end{array}$$

Based on the above analysis and setting, the following objective function is constructed to obtain an optimal weight vector that can maximize the deviation value of overall scores of all doctors for each expert evaluation.

(17) $$\begin{array}{l} D\left(x\right)=\overset{K}{\underset{K=1}{\sum }}D_{k}\left(x\right)=\overset{K}{\underset{K=1}{\sum }}\overset{N}{\underset{i=1}{\sum }}\overset{N}{\underset{t=1}{\sum }}|s\left(h_{ki}\right)w_{k}-s\left(h_{kt}\right)w_{k}| \end{array}$$

To solve the weight vector, the following model and Lagrange function are constructed:

(18) $$\begin{array}{l} \mathrm{maxD}\left(w\right)=\max\left\{\overset{K}{\underset{K=1}{\sum }}\overset{N}{\underset{i=1}{\sum }}\overset{N}{\underset{t=1}{\sum }}|s\left(h_{ki}\right)w_{k}-s\left(h_{kt}\right)w_{k}|\right\} \end{array}$$

(19) $$\begin{array}{l} s.t\{\begin{array}{cc} \overset{k}{\underset{k=1}{\sum }}{\left(w_{k}\right)}^{2}=1\\ w_{k}\geq 0, & k=1,2,\ldots ,k \end{array} \end{array}$$

(20) $$\begin{array}{l} L\left(w,\eta \right)=\overset{K}{\underset{K=1}{\sum }}\overset{N}{\underset{i=1}{\sum }}\overset{N}{\underset{t=1}{\sum }}|s\left(h_{ki}\right)w_{k}-s\left(h_{kt}\right)|w_{k}+\frac{\eta }{2}\left(\overset{k}{\underset{k=1}{\sum }}{\left(w_{k}\right)}^{2}=1\right) \end{array}$$

Combined with the above formula, we can get the following:

(21) $$\begin{array}{l} \bar{w_{k}}=\frac{\overset{N}{\underset{i=1}{\sum }}\overset{N}{\underset{t=1}{\sum }}|s\left(h_{ki}\right)w_{k}-s\left(h_{kt}\right)w_{k}|}{\overset{K}{\underset{K=1}{\sum }}\overset{N}{\underset{i=1}{\sum }}\overset{N}{\underset{t=1}{\sum }}|s\left(h_{ki}\right)w_{k}-s\left(h_{kt}\right)w_{k}|} \end{array}$$

According to the expert advice and MSD method, the optimal weights of each step in the similarity calculation can be obtained.

### Calculation and Update of Credit Line

#### Aggregation of Historical Records

In the previous section, we calculated a value describing the behavior of doctors, correlation.

The historical record of each doctor is composed of calculated correlations. In a period, the doctor will generate a large number of historical records. When calculating the credit limit, the historical records are summarized according to the timeline. In the process of calculating and updating the credit limit, the time recorded in history is the time when each doctor diagnosed a certain patient. The influence of early historical records on credit lines will diminish over time. On the contrary, if the behavior of curious doctors occurs recently, the impact on credit will be even greater. As a penalty, the credit limit will remain low for a period.

Since the historical visit record is composed of similarity and time window, the value of similarity is a percentage, in the range of [0,1], so there is no need for standardization. However, the time of each historical access record needs to be mapped in the range of [0,1], that is, the data are standardized. Suppose the time window before processing is *A* = (a_1_, a_2_, …, a_n_), and the time window after standardization is *B* = (b_1_, b_2_, …, b_n_).

The mapping method is as follows:

(22) $$\begin{array}{l} B_{\text{i}}=\{\begin{array}{cc} \frac{a_{i}-{\left(a_{i}\right)}_{\min}}{{\left(a_{i}\right)}_{\max}-{\left(a_{i}\right)}_{\min}}, & a_{i}>0\\ \frac{{\left(a_{i}\right)}_{\max}-a_{i}}{{\left(a_{i}\right)}_{\max}-{\left(a_{i}\right)}_{\min}}, & a_{i}<0 \end{array} \end{array}$$

To make the calculation of credit limit more objective and authentic, the earlier historical record in real life will have less impact on the current credit, that is, the longer the historical record is, its value will decay over time. Suppose the set *HT* = {*T*_*hk*_(1 ≤ *k* ≤ *q*)}(*q* = |*HT*|) of medical history records and the corresponding time window *B* = {*b*_*k*_|1 ≤ *k* ≤ *q*}, then the time attenuation function for a task *h*_*k*_(1 ≤ *k* ≤ *q*) is as follows:

(23) $$\begin{array}{l} \phi \left(t\right)=\frac{1-b_{k}/\sum_{k=1}^{q}b_{k}}{\overset{q}{\underset{k=1}{\sum }}\left(1-b_{k}/\sum_{k=1}^{q}b_{k}\right)} \end{array}$$

**Figure 3:**
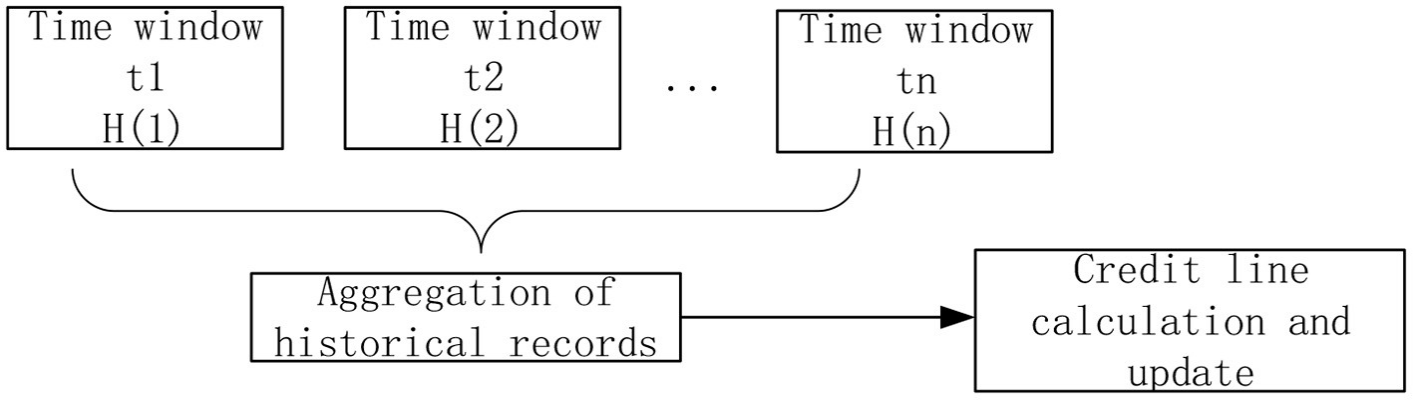
History aggregation.

When calculating the credit limit, the model proposed by Caverlee et al. (44) is modified. Each history record is distinguished by a time window. The structure diagram of the aggregate value calculated according to the historical record of the user in the past N cycles is shown in the Figure 3:

The aggregate calculation formula for history of a user *H*(1)…*H*(*n*) in the past N cycles is as follows:

(24) $$\begin{array}{l} H\left(old\right)=\frac{1}{\gamma }\times \overset{N}{\underset{k=1}{\sum }}H_{K}\times \alpha^{N-K} \end{array}$$

wherein $\gamma =\overset{N}{\underset{k=1}{\sum }}\alpha^{N-K}$, γ is used to limit the credit value obtained after aggregation to remain within the original credit value range; α is the adjusting parameter of the influence of historical records to the current trust evaluation. The value range of α is 0 < α < 1, the smaller the α is, the less important the historical record is.

The updated formula of the credit limit can be obtained based on the aggregate results of the above historical records:

(25) $$\begin{array}{l} H_{\text{new}}=\{\begin{array}{cc} H_{\text{old}}\cdot \left[1+\varphi \left(\Delta H\right)\right], & t>t_{0}\\ H_{old}\cdot \phi \left(t\right), & t<t_{0} \end{array} \end{array}$$

*H*_old_ represents the initial line of credit that is aggregated according to the historical records for the first time, *H*_new_ represents the value of the line of credit after constant updates, t_0_represents the effective time of the set time window, and the time decay function. When the time interval t is less than t_0_, it means that the current operation occurs within the same time window as the last one. At this time, the credit line is not updated, and the time decay function is used for processing. When t is greater than t_0_, it means that within the next time window, the new aggregate value and the increment Δ*H* = *H*_new_ − *H*_*old*_ of the historical aggregate value are used to recalculate and update the value of the credit line.

## Dynamic Access Control Based on Trust

### Overview

In this study, the concept of the credit line is introduced to improve the access control of the consultation process of doctors in the existing intelligent medical system. The doctor logs in HIS according to the identity information (the doctor logs in the device, time, place, etc.), and each user calculates the corresponding credit limit according to the system, which is used to match the reasonable permissions according to the access control strategy.

After the doctor finishes each diagnosis, the data such as the visit record from HIS will be saved in the historical record. Through trust calculation, the credit limit of the user within a period can be obtained.

**Figure 4:**
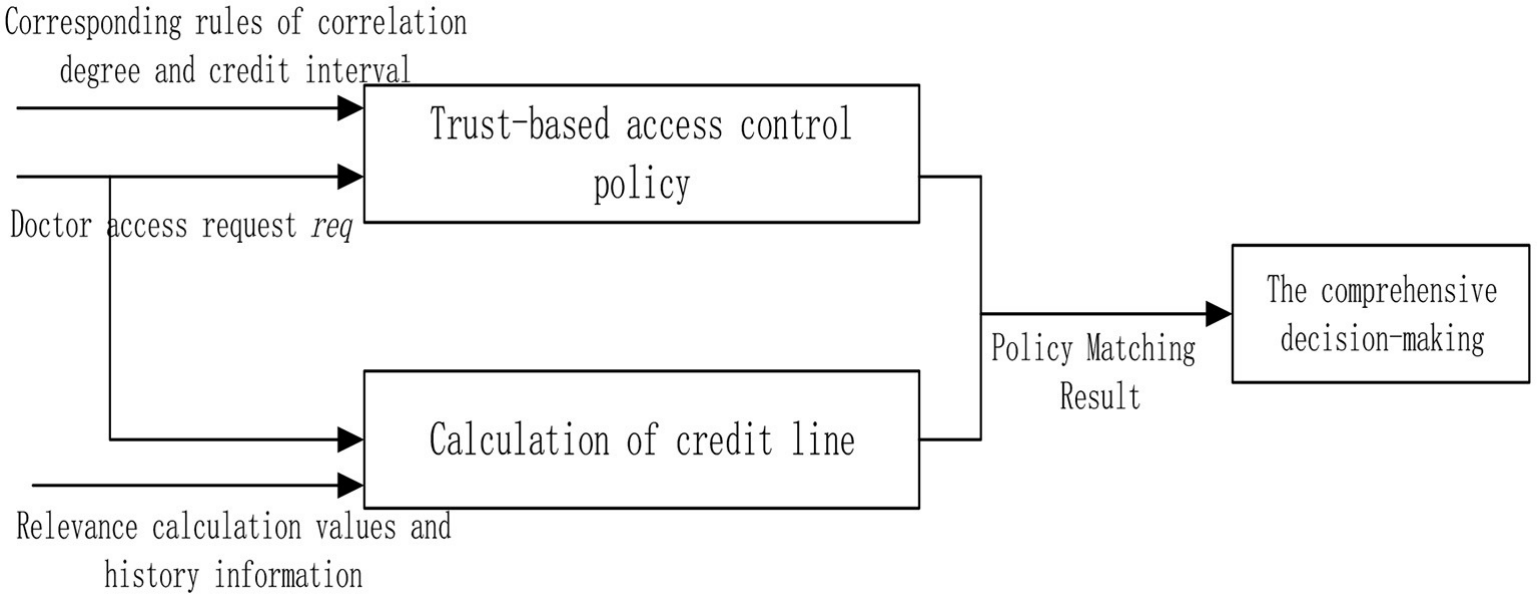
Access control scheme.

The trust interval corresponds to the degree of openness of the permission. For example, the line of credit of a doctor is t (t2 < t ≤ t3). According to the access control strategy, the doctor is only allowed to visit contents with relevance of 0.6 during the diagnosis and treatment process. If the doctor visits too many irrelevant contents, the decline of the operational relevance will lead to the reduction of the credit limit of the doctor. The access request of the user is denied when the amount is insufficient. The credit interval of the proposed scheme and overall flow chart is shown in the Figure 4.

### Access Control Policies

**Figure 5:**
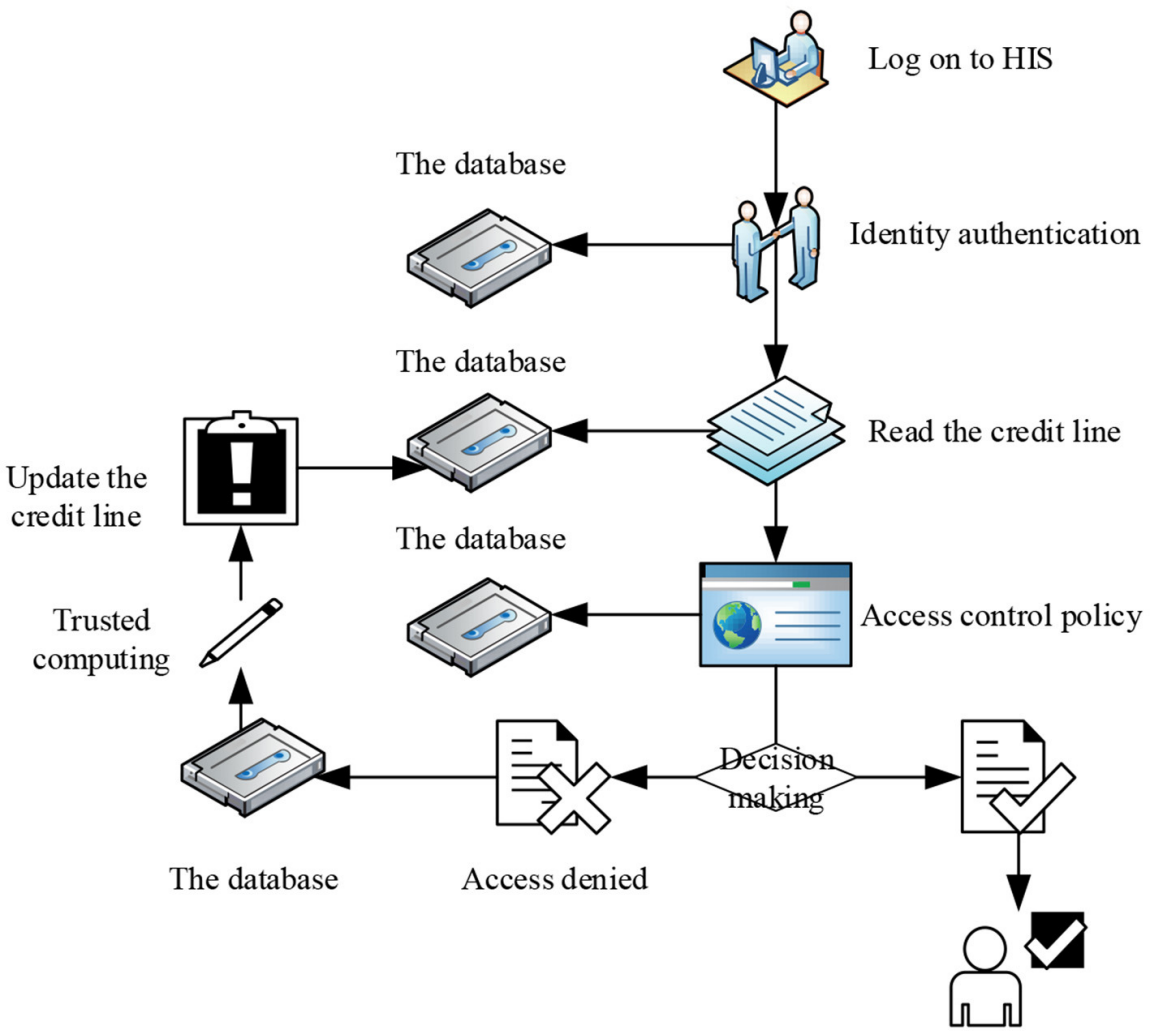
Access control flow chart.

An access control policy is the key point of the access control model, which is the access rule set and condition constraint set of subject to object. In the background of the HIS system in this study, the subject is set as the doctor, and the object is the medical record. The access control flow for this article is shown in Figure 5.

Step 1. Read the trust value of the doctor from the library and compare the threshold t_0_(DT_i_ϵDT,t_0_ < t ≤ t_4_). The value range of T is shown in Table 1.

**Table 1:** Correlation degree—credit interval rules.

	**Correlation**	**The line of credit is in the range**
1	0.8	t_3_ < t ≤ t_4_
2	0.6	t_2_ < t ≤ t_3_
3	0.4	t_1_ < t ≤ t_2_
4	0.2	t_0_ < t ≤ t_1_

(a) if(DT_i_.t < t_0_)return false;

If credit limit of the doctor is below the threshold value t_0_, the decision to deny access request is returned and recorded.

(b) if(DT_i_.t≥t_0_)

When credit line of the doctor is higher than the threshold value t_0_, proceed to Step 2.

Step 2. Match the trust interval according to credit limit of the doctor:

(c) if (t_0_ < DT_i_.t ≤ t_1_) trust = 1;

else if (t_1_ < DT_i_.t ≤ t_2_) trust = 2;

else if (t_2_ < DT_i_.t ≤ t_3_) trust = 3;

else trust = 4;

If the trust value of the doctor belongs to (t_0_, t_1_), then 1 is returned, indicating that access rights belong to level 1.

If the trust value of the doctor is (t_1_, t_2_), then return 2, indicating that the access is level 2.

If the trust value of the doctor is (t_2_, t_3_), then return 3, which means that the access is level 3.

If the trust value of the doctor belongs to (t_3_, t_4_), then 4 is returned, representing that the access authority belongs to level 4.

Step 3. Match the corresponding relevance requirements according to the credit interval.

Switch(trust)

{

case 1 : pre(S)=0.9;

case 2 : pre(S)=0.6;

case 3 : pre(S)=0.4;

case 4 : pre(S)=0.2;

}

pre (S) specifies the minimum value of the access relevancy. If it is lower than this value, it will be reflected in the historical record, which will greatly affect the next round of credit evaluation.

## Experimental Analysis

### Data Sources

Relying on the project of the National Natural Science Foundation of China, this study completed relevant research experiments according to the medical data set provided by a third-class hospital of Kunming, the cooperative unit of the project. The data set contains rich text data and image data, with a total of five databases, the size of which is 1,200 G, including 1,360 data tables and a total of 21,39,373 records. In this experiment, part of medical data was extracted to simulate visits of doctors in the process of diagnosis and treatment.

### Experimental Settings

The purpose of the experiment is to verify whether the access control model based on HIS proposed in this study can calculate the line of credit through the historical behavior records of doctors, and well control the access rights of doctors through the value of the line of credit. The data of HIS account access records of three doctors in a department provided by the cooperative hospital were selected for calculation, including one doctor who simulated the behavior of a curious doctor and one honest doctor who simulated a special visit situation as the experimental group.

### Weights

Ten medical experts were asked to directly give the weights of the relevant calculations. The weights calculated by the MSD method according to Equation (21) are shown in the Table 2. To verify whether the weight calculated according to the weight calculation method, MSD, is better than the weight directly given by the expert, randomly select the weights of two groups of experts and the weights calculated by the MSD method for comparison experiments.

**Table 2:** Expert opinion weights.

	**α**	**β**	**γ**
Expert 1	0.6	0.2	0.2
Expert 2	0.3	0.6	0.1
Expert 3	0.5	0.3	0.2
…	…	…	…
Expert 9	0.4	0.3	0.3
Expert 10	0.2	0.3	0.5
MSD calculate	0.49	0.16	0.35

The three doctors are honest doctors, non-malicious doctors with special circumstances (hereinafter referred to as special doctors), and curious doctors. In HIS, each doctor completed 15 diagnoses, among which the curious doctor completed three malicious behaviors, and the special doctor completed two special case diagnoses.

**Figure 6:**
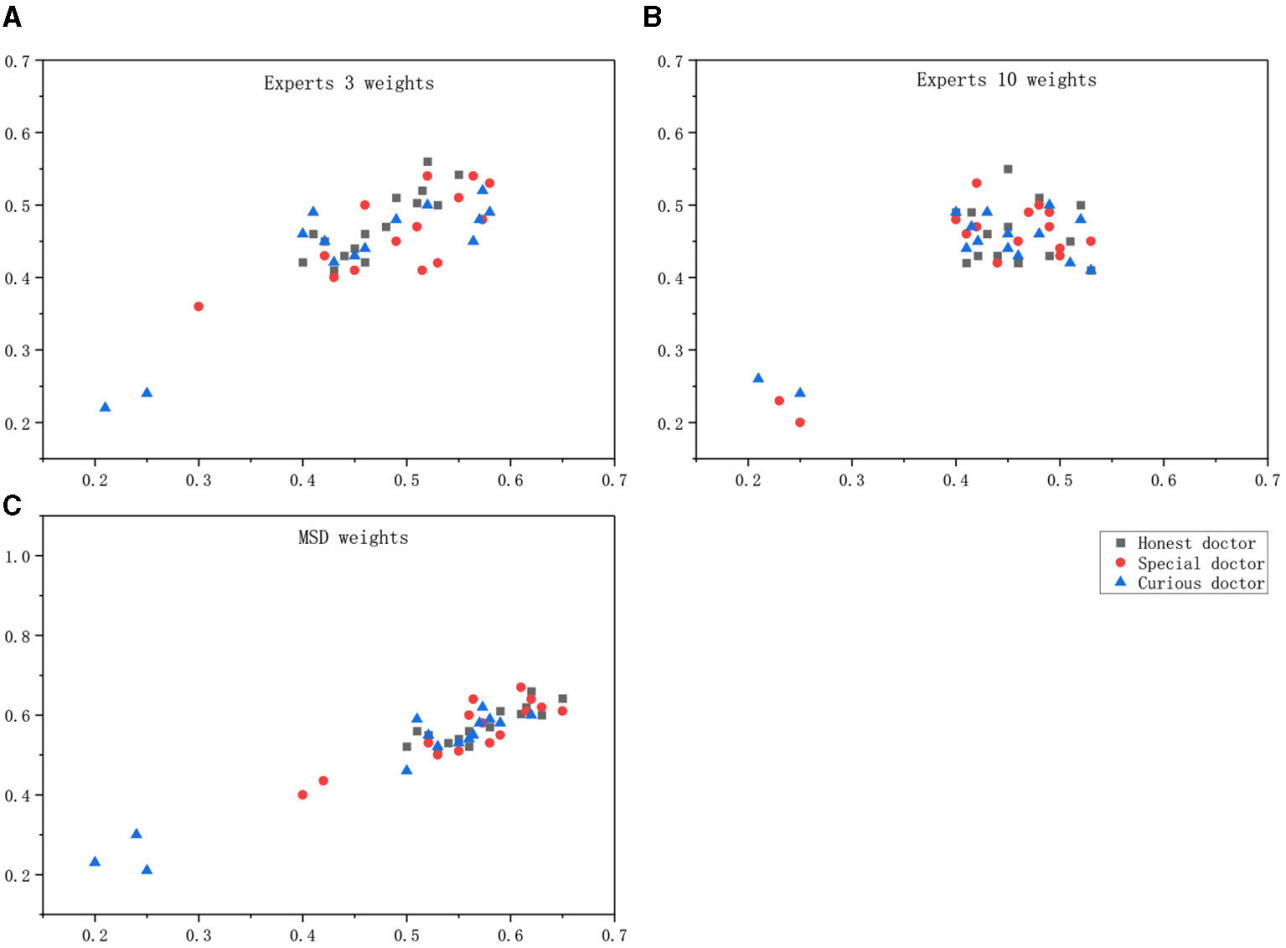
Maximum score deviation (MSD) weight differentiating doctor effect. **(A)** Experts 3 weights, **(B)** Experts 10 weights, **(C)** MSD weights.

In addition, weights were set according to the weights directly given by Expert 3 and Expert 10 as well as the MSD calculation results, and the scatterplot drawn could intuitively see the calculation results of the correlation degree as shown in the Figure 6.

Maximum score deviation weights can distinguish between malicious behavior, special behavior, and normal behavior.

According to the obtained images, the analysis in the following Table 3 can be obtained. The correlation calculated by the weight given by an expert alone cannot make an accurate judgment on the behavior of doctors, especially in the discrimination between curious doctor and special doctor.

**Table 3:** The results of doctors were judged by maximum score deviation (MSD) weights.

		**Expert 3**			**Expert 10**			**MSD**		
		**Step 1**	**Step 2**	**Step 3**	**Step 1**	**Step 2**	**Step 3**	**Step 1**	**Step 2**	**Step 3**
Special access	1	°			×			°		
	2	•			×			°		
Malicious access	1	°			•			°		
	2		°			°			°	
	3			•			°			°

*•, Unable to determine; °, right; ×, false*.

### Aggregation

According to Equation (25), with a period of 1 month, the aggregate value of historical records is used to calculate the changes in the credit lines of the three types of doctors in eight periods, as shown in the following Table 4:

**Table 4:** Changes of historical aggregate values of doctors.

**Doctor**	**CT(1)**	**CT(2)**	**CT(3)**	**CT(4)**	**CT(5)**	**CT(6)**	**CT(7)**	**CT(8)**
Honest doctor	0.66	0.57	0.62	0.59	0.67	0.63	0.60	0.59
Special doctor	0.63	0.60	0.58	0.56	0.64	0.51	0.61	0.56
Curious doctor	0.51	0.43	0.57	0.64	0.66	0.67	0.58	0.47

According to the calculation, the average value of the credit line in eight periods is obtained as Table 5.

**Table 5:** The variation of the mean value of credit line with α.

**Doctor**	**Δ**	**α = 0.95**	**Δ**	**α = 0.5**	**Δ**	**α = 0.2**	**< Δ**
Honest doctor	0.16	0.596	0.11	0.604	0.12	0.583	0.13
Special doctor	0.31	0.557	0.12	0.585	0.14	0.602	0.19
Curious doctor	0.34	0.531	0.26	0.538	0.17	0.551	0.25

Three historical record influencing parameters α were given: 0.95, 0.5, and 0.2, and the credit limit of three kinds of doctors was calculated based on the aggregation of historical records in eight cycles. In the table, Δ represents the maximum fluctuation range of the line of credit under the corresponding value of α. Column $\bar{\Delta }$ records the mean fluctuation range of the line of credit.

**Figure 7:**
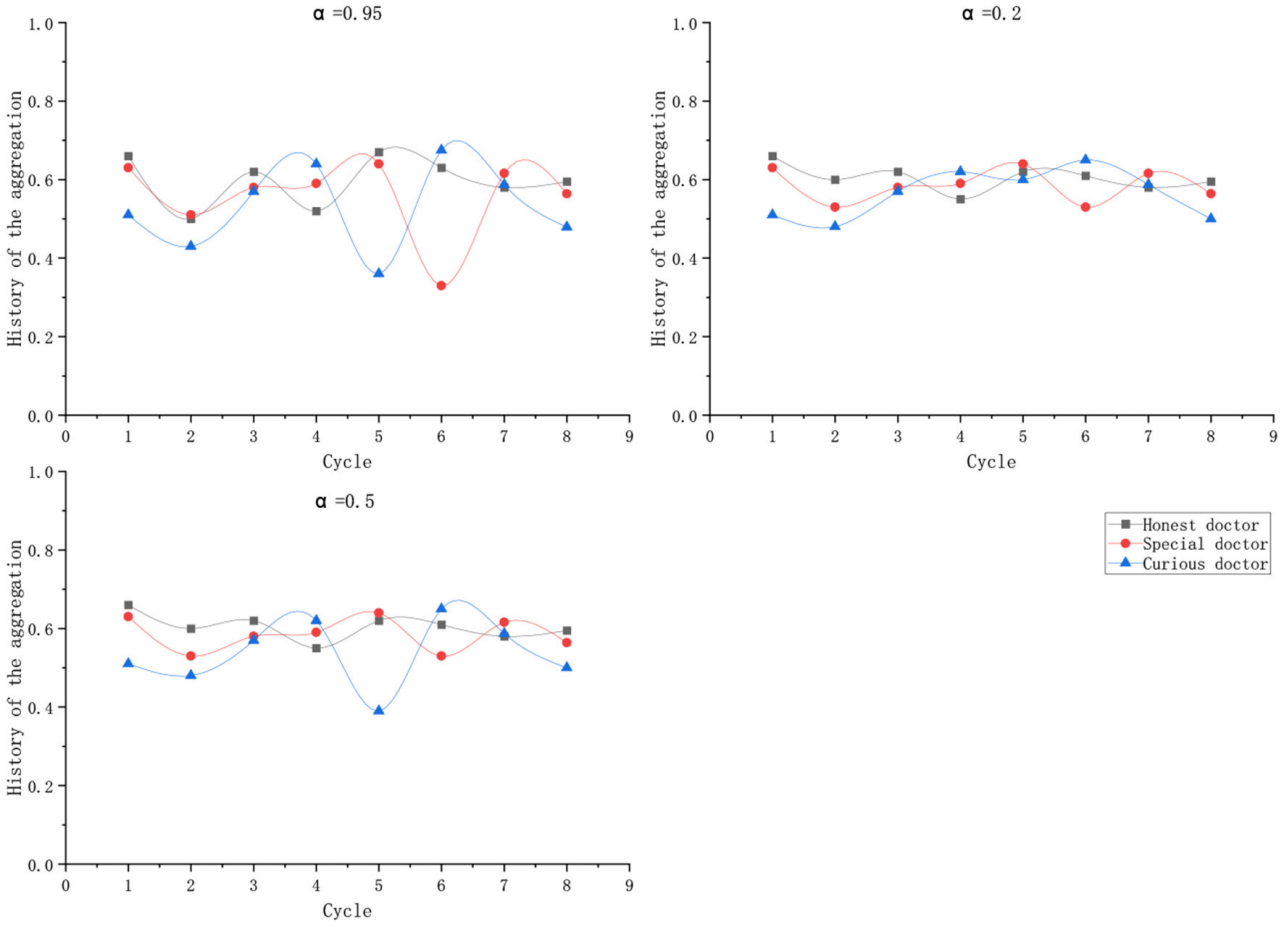
Fluctuation of credit line.

As shown in the Figure 7, an appropriate α can keep the credit limit of doctors who maintain normal behavior during the diagnosis process in a relatively stable state, but they are sensitive to the malicious behavior of curious doctors.

### Access Control Experiment

**Figure 8:**
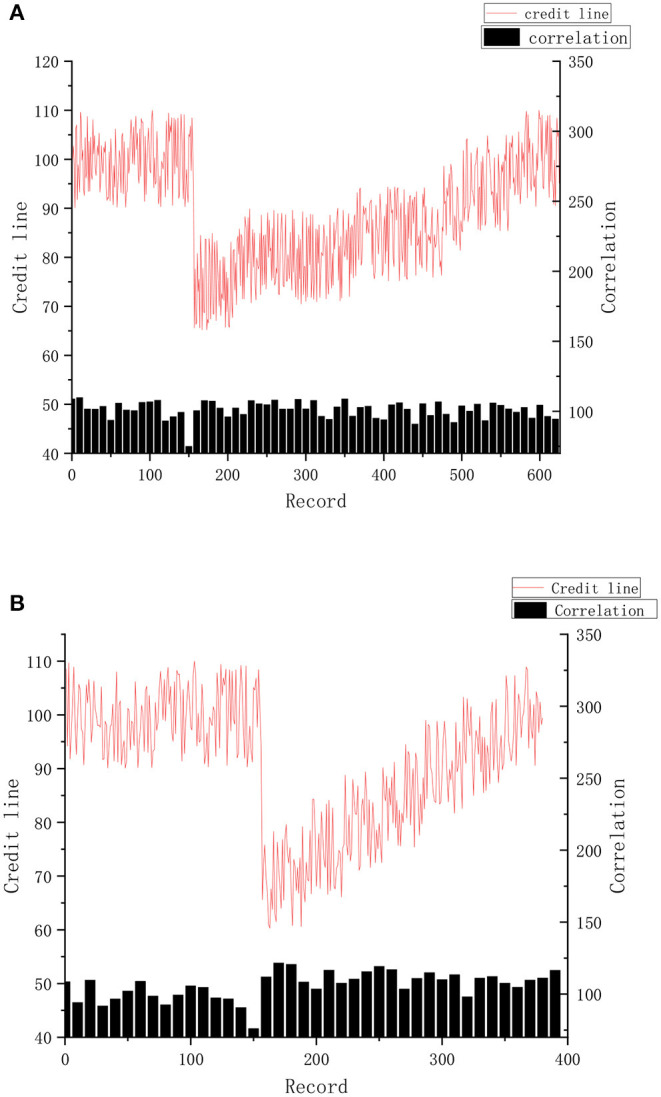
**(A,B)** Change of credit limit and correlation degree.

The period N of the historical record for the calculation of the credit limit was 1 month. Assuming that each doctor arranges 3 days a week to diagnose patients, the average daily medical record is about 50. According to the results of the experiment, when malicious visit of the doctor occurred, the credit line completely returned and stabilized at the original level, which required about 650 records, which took nearly a month. This situation is shown in Figure 8A.

If doctors intend to increase their average interview relevancy after malicious visits, as shown in the experimental results in the Figure 8B, it requires about 250 records, nearly half a month, to completely stabilize the original level of the credit line.

The experiment proves that when malicious access occurs, the value of the credit limit will be immediately affected. As punishment for privacy risk, the credit limit will be kept at a low value for a long period to warn users of their bad behavior and achieve the effect of access control at the same time.

### Contrast Experiment

The hospitals that our project cooperates with are currently using traditional HIS without access control. Hundred doctors from the hospital were randomly selected for a black-box test. The doctors were divided into two groups, and the traditional HIS and the HIS of the trusted access control model proposed in this study were used for a 1 month comparison test. In the case that the doctor does not know the contents of the experiment, the historical records of the two groups of doctors are analyzed.

**Figure 9:**
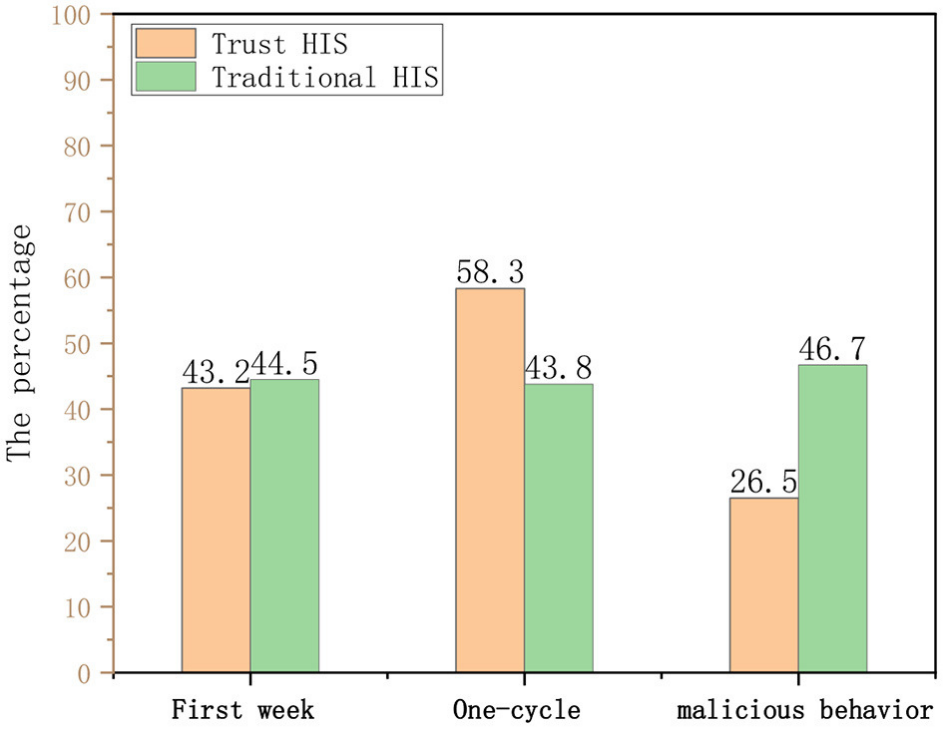
Comparison of health information system (HIS) correlation degree and behavioral constraint ability.

It can be seen from the Figure 9 that there is no significant difference in the historical visit records (correlation) of the doctors using the two HISs within 1 week of the experiment. Throughout the experimental cycle, the relevance of doctors using traditional HIS has not changed significantly, while the trust HIS model has been significantly improved, indicating that the proposed credit line can regulate user behavior to a certain extent.

Then, we conducted a questionnaire survey of some doctors in the hospital. The purpose is to compare the credit line model proposed in this study with the role-based access control model (hereinafter referred to as role) and ABAC model (hereinafter referred to as attribute). The feedback from all 256 users is shown in the Table 6.

**Table 6:** User evaluation table of doctors.

	**Difficulty of malicious access**			**Risk of rejection of special access requests**			**Degree of system automation**		
Level	I	II	III	I	II	III	I	II	III
Attribute	73	128	55	42	189	25	16	127	13
Role	156	56	44	206	34	16	116	25	15
Trust	80	84	92	10	34	212	3	64	189

**Figure 10:**
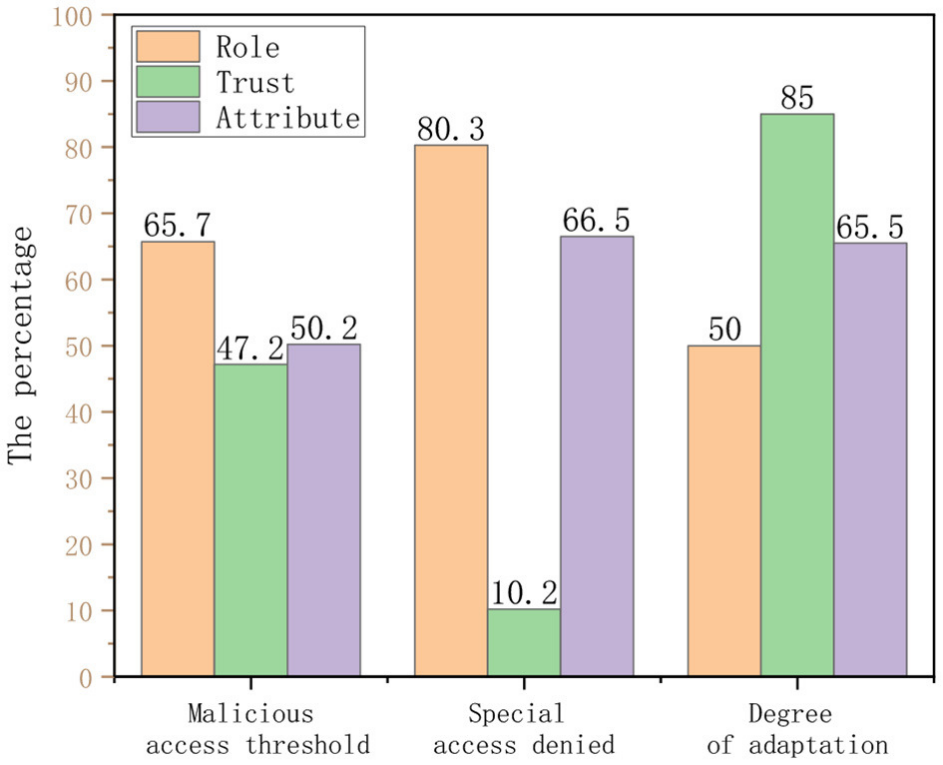
Traditional HIS vs. trust-based access control HIS.

According to the table data and Figure 10, the following chart shows that the trust-based HIS access control model proposed in this study has a good performance in terms of flexibility of access control, preventing malicious access behavior from occurring, and the degree of system automation.

## Conclusion

Aiming at HIS in the context of medical big data, this study proposes a dynamic access control model for doctors in the process of diagnosis and treatment. First, according to the diagnosis and treatment process of the doctor, the behavior model of the doctor is designed, and three hypotheses of privacy leakage are proposed. Then, according to the operation correlation of the doctor, time index, and other factors, the behavior of the doctor in the diagnosis and treatment process is described, and the purpose is to calculate the rationality of the diagnosis process of the doctor through mathematical methods. Finally, by calculating the credit limit, the access control strategy using the credit limit interval dynamically restricts the access ability of the doctor in the diagnosis and treatment process. Experiments prove that the model designed in this study can accurately identify bad doctors and inhibit their visits by trust value, and the ability to prevent patient privacy leakage is better than traditional HIS.

## Data Availability Statement

The raw data supporting the conclusions of this article will be made available by the authors, without undue reservation.

## Author Contributions

RJ proposed the idea for this paper. RJ, WW, and YY designed the study. WW wrote the paper. WW and FM performed the experimental analysis. All authors reviewed and edited the manuscript and read and approved the manuscript.

## Funding

This work was supported by the National Natural Science Foundation of China (Nos. 71972165, 61763048), Science and Technology Foundation of Yunnan Province (No. 202001AS070031).

## Conflict of Interest

The authors declare that the research was conducted in the absence of any commercial or financial relationships that could be construed as a potential conflict of interest.

## Publisher's Note

All claims expressed in this article are solely those of the authors and do not necessarily represent those of their affiliated organizations, or those of the publisher, the editors and the reviewers. Any product that may be evaluated in this article, or claim that may be made by its manufacturer, is not guaranteed or endorsed by the publisher.
